# Interleukin-1beta and tumor necrosis factor-alpha are expressed by different subsets of microglia and macrophages after ischemic stroke in mice

**DOI:** 10.1186/1742-2094-5-46

**Published:** 2008-10-23

**Authors:** Bettina H Clausen, Kate L Lambertsen, Alicia A Babcock, Thomas H Holm, Frederik Dagnaes-Hansen, Bente Finsen

**Affiliations:** 1 Medical Biotechnology Center, University of Southern Denmark, Odense, Denmark; 2 Department of Medical Microbiology and Immunology, University of Aarhus, Aarhus, Denmark

## Abstract

**Background:**

Interleukin-1β (IL-1β) and tumor necrosis factor-α (TNF-α) are expressed by microglia and infiltrating macrophages following ischemic stroke. Whereas IL-1β is primarily neurotoxic in ischemic stroke, TNF-α may have neurotoxic and/or neuroprotective effects. We investigated whether IL-1β and TNF-α are synthesized by overlapping or segregated populations of cells after ischemic stroke in mice.

**Methods:**

We used flow cytometry and immunohistochemistry to examine cellular co-expression of IL-1β and TNF-α at 6, 12 and 24 hours after permanent middle cerebral artery occlusion in mice, validating the results by the use of bone marrow chimeric mice.

**Results:**

We found that IL-1β and TNF-α were expressed in largely segregated populations of CD11b^+^CD45^dim ^microglia and CD11b^+^CD45^high ^macrophages, with cells expressing both cytokines only rarely. The number of Gr1^+ ^granulocytes producing IL-1β or TNF-α was very low, and we observed no IL-1β- or TNF-α-expressing T cells or astrocytes.

**Conclusion:**

Taken together, the results show that IL-1β and TNF-α are produced by largely segregated populations of microglia and macrophages after ischemic stroke in mice. Our findings provide evidence of a functional diversity among different subsets of microglia and macrophages that is potentially relevant to future design of anti-inflammatory therapies in stroke.

## Background

The proinflammatory cytokines interleukin-1β (IL-1β) and tumor necrosis factor-α (TNF-α) play key roles in the pathogenesis of ischemic stroke [1-3]. IL-1β exerts neurotoxic effects in ischemic stroke and blocking its action has been shown to reduce ischemic brain damage [4,5]. In comparison, there is evidence that TNF-α has both neurotoxic [6,7] and neuroprotective [8-10] roles after ischemic stroke in rats and in mice. Increasing evidence implicates both cytokines in the early inflammatory response that precedes and accompanies ischemia-induced neuronal damage [6,11]. However, detailed knowledge about the contribution of different cell types to the production of IL-1β and TNF-α is still not available.

The relative physiological outcome of increased IL-1β and TNF-α signaling in ischemic stroke may depend on the kinetics and location of cytokine producing cells. There is compelling evidence that IL-1β and TNF-α are primarily synthesized by activated microglia and infiltrating macrophages [12-14], although granulocytes and astrocytes have also been suggested to produce both IL-1β [15-17] and TNF-α [18,19]. Precise identification of cell source has, however, been compromised by the lack of microglial and macrophage specific markers, which prevents discrimination of these cell types at the histological level [12,14]. Furthermore, it is presently unknown whether IL-1β and TNF-α are expressed to the same extent by the same or different subsets of microglia and macrophages following ischemic stroke.

We have previously shown that IL-1β mRNA and TNF-α mRNA, and TNF-α protein are produced by CD11b^+ ^microglia and by CD11b^+ ^macrophages at the edge of and within areas of infarction, and that this production reaches maximum levels of expression between 12 and 24 hours after permanent middle cerebral artery occlusion (pMCAO) in mice [12,14,20]. The objective of the present study was to provide additional insight into the cell types and cell subpopulations that produce IL-1β and TNF-α within the first 24 hours following ischemic stroke in mice [20]. To distinguish microglia from infiltrating macrophages after pMCAO, we used flow cytometry with CD45 and CD11b as myeloid-lineage specific markers, we used a radiated, bone marrow (BM) chimeric mouse model; and we used intracellular cytokine-staining, and double immunofluorescence staining. In addition, since CD11b is expressed by both macrophages and granulocytes [21,22], we also analyzed cytokine production by granulocytes using the granulocyte specific marker Gr1. Our results show that IL-1β and TNF-α are produced by largely segregated subsets of microglia and macrophages, and that very few cells express both cytokines.

## Methods

### Animals

Breeding pairs of CD45.1^+ ^(B6.SJL-Ptprc^a ^Pepc^b^/BoyJ) mice and CD45.2^+ ^C57BL/6-Tg(UBC-GFP)30Scha/J (GFP-Tg) [23] mice were purchased from the Jackson Laboratory (Bar Harbour, Maine, USA) and transferred to the Department of Medical Microbiology and Immunology, University of Aarhus, where they were maintained as a colony. GFP-Tg mice, which express the CD45.2^+ ^allotype, were used as BM donors and congenic BoyJ male mice, which express the CD45.1^+ ^allotype, were used as BM recipients. This combination of mice was chosen so that infiltrating cells could be identified by two different markers (GFP^+ ^and CD45.2^+^), however the GFP signal alone proved to be sufficiently strong and reliable [24] to identify infiltrating cells. Peritoneal macrophages were obtained from C57BL/6 mice, which were purchased from Taconic (Ry, Denmark). Mice were housed under diurnal lightning conditions with free access to food and water. The experiments were approved by the Danish Animal Inspectorate (J. no. 2005/561–1068).

### Generation of bone marrow chimeras

BM cells from GFP-Tg mice were grafted into lethally irradiated mice as described by Wirenfeldt et al. [24]. For donor BM recovery, the proximal and distal ends of tibia and femur were removed and BM was flushed from the medullary channel into sterile 50 mL polypropylene tubes using cold RPMI 1640 medium (Gibco, Paisley, UK). The BM cells were rinsed in RPMI medium and centrifuged at 1,000 rpm for 10 min, then filtered through a sterile cotton sieve. After 3 rounds of rinsing and filtering, cells were kept on ice until use. Recipient mice (6–8 weeks of age) were lethally irradiated with a single dose of 9.5 Gy from a ^137^Cs source (Risø National Laboratory, Roskilde, Denmark). Within 2 hours of irradiation, BM cells were injected into the tail veins of recipient mice (approximately 10^7 ^cells/mouse). For 3 days after grafting, mice received oxytetracycline (2 g/L Terramycin vet. 20%; Pfizer, Amoise, France) in autoclaved drinking water. Thereafter they were supplied with acidified water (pH 3). The mice were fed irradiation-sterilized mouse chow and maintained under pathogen-free conditions for 6 weeks until they were subjected to surgery.

### Permanent middle cerebral artery occlusion

Under anaesthesia, mice were subjected to focal cerebral ischemia by permanent occlusion of the distal part of the left middle cerebral artery (pMCAO), as previously described [20]. During surgery, mice were placed on a 37°C ± 0.5°C warm heating pad. An incision was made from eye to ear, the parotid gland and the upper part of the temporal muscle were split, and a small hole was drilled over the distal part of the MCA. The MCA was occluded by electrocoagulation and the incision was stitched with a 4.0 nylon-suture. After surgery, mice were injected with 1 ml of isotonic saline and their eyes were coated with ointment. Mice were kept at 28°C until sacrificed, 24 hours post-surgery. For post-surgical analgesia mice were treated with Temgesic (0.001 mg/20 g buprenorphinum, Reckitt & Colman, Hull, UK) three times at 8-hour intervals starting immediately after surgery [20].

### Tissue preparation

#### Histology

Mice were anesthetized i.p. with an overdose of pentobarbital and perfused through the left ventricle using 10 ml of Soerensen's phosphate buffer (SB) (0.03 M KH_2_PO_4 _and 0.12 M Na_2_HPO_4_, pH 7.4) followed by 20 ml of cold 4% paraformaldehyde (PFA) in 0.15 M SB, pH 7.4. The brains were post-fixed in 4% PFA for 1 hour followed by immersion in 20% sucrose in 0.15 M SB overnight. Next, the brains were frozen and cut into 16-μm frontal sections. For verification of chimerism in BM-chimeric mice, samples of spleen, heart, kidney and liver were frozen and processed to obtain 16-μm cryostat sections.

#### Cell culture preparation

Thioglycolate-elicited peritoneal macrophages were obtained after i.p. injection of 1 ml 3% thioglycolate (Sigma) into C57BL/6 mice. Three days after injection, the mice were killed by cervical dislocation and 10 ml Dulbecco's modified Eagle Medium (Invitrogen) was injected into the peritoneum. Subsequently, the peritoneal fluid was withdrawn. After two washes in phosphate buffered saline (PBS, pH 7.4) the cells were counted and plated in T25 flasks in DMEM Medium containing 5% FBS, 100 units/ml of penicillin, and 100 mg/ml streptomycin (Sigma). The macrophages were kept in a humidified 37°C incubator with 5% CO_2 _for 2 days and then stimulated for 24 hours with 100 ng/ml LPS (Sigma).

#### Flow cytometry

Mice were perfused through the left ventricle using 10 ml PBS, whereafter the ipsi- and contralateral cortices were isolated. Infarcts were macroscopically visible at 6, 12 and 24 hours post-occlusion, verifying successful occlusion of the MCA. Blood samples were collected in heparinized Eppendorf tubes and Hanks' balanced salt solution (HBSS: 0.14 M NaCl, 5.4 mM KCl, 0.4 mM MgSO_4_•7H_2_O, 0.4 mM Na_2_HPO_4_(anhydrate), 1.3 mM CaCl_2_2H_2_O, 4.2 mM NaHCO_3_, 0.4 mM KH_2_PO_4_, 0.5 mM MgCl_2_6H_2_O, and 5 mM glucose) for validation of BM reconstitution. Lymph nodes were removed and collected as control tissue.

### Immunohistochemistry and cell counting

#### Immunohistochemistry for CD11b, CD45, IL-1β and TNF-α

Microglial/macrophage staining by CD11b or CD45 was done using an streptavidin/horseradish peroxidase technique as previously described [20]. Immunohistochemical stainng for IL-1β and TNF-α was performed as previously described [12,14] (Table 1).

**Table 1:** Antibodies applied for flow cytometry, immunohistochemistry and fluorescence stainings.

**Cell population**	**Antibody**	**Isotype**	**Species of origin**	**Cat. #**
Flow cytometry:				
T-cells	TCRβ-APC	IgG2	Hamster	553174, BD Biosciences*
Granulocytes	GR1-FITC	IgG2b	Rat	553126, BD Biosciences
TNF producing cells	TNF-PE	IgG1	Hamster	559503, BD Biosciences
IL-1β producing cells	IL-1β		Rabbit	AAM13G, Serotec
Microglia/macrophages	CD11b-PE/-APC/-PerCP-Cy5.5	IgG2b	Rat	557397/553312/550993, BD*
Microglia/macrophages	CD45-PE/-APC/-PerCP-Cy5.5	IgG2b	Rat	553081/559864/550994, BD*
Isotype/serum controls:	PE-/APC-/PerCP-Cy5.5-conjugated	IgG2b	Rat	553989/556924/550764, BD*
	FITC-conjugated	IgG2b	Rat	556923, BD Biosciences
	PE-conjugated	IgG1	Hamster	554711, BD Biosciences
	APC-conjugated	IgG2	Hamster	558141, BC Biosciences
	Un-conjugated	Ig fraction	Rabbit	X0903, DakoCytomation
**Secondary antibodies:**				

Anti-rabbit I	Alexa Fluor647-conjugated	IgG	Goat	A21244, Invitrogen
Anti-rabbit II	Alexa Fluor488-conjugated	IgG	Chicken	A21441, Invitrogen
				
Immunohistochemistry:				
BM-derived cells	GFP (1:1000)		Rabbit	ab290, Abcam
TNF producing cells	TNF (1:50)		Rat	MM-350, Endogen
TNF producing cells	TNF (1:200)		Rabbit	P-350, Endogen
IL-1β producing cells	IL-1β (1:50)		Rabbit	AAM13G, Serotec
Microglia/macrophages	CD11b (1:600)	IgG2b	Rat	MCA711, Serotec
	CD45.1 Biotin-conjugated (1:80)	IgG2a,κ	Mouse	553774, BD Biosciences
	CD45.2 Biotin-conjugated (1:80)	IgG2a,κ	Mouse	553771, BD Biosciences
	CD45 (1:80)	IgG1	Rat	MCA1388, Serotec
Granulocytes	Ly-6G and Ly-6C(Gr1) (1:200)	IgG2b	Rat	01211A, BD Biosciences
Endothelial	von Willebrandt's Factor (1:200)		Rabbit	A0082, DakoCytomation
Endothelial	PECAM-1/CD31 (1:100)	IgG2a	Rat	557355, BD Biosciences
Astrocytes	GFAP (1:1,200)		Rabbit	Z0334, DakoCytomation
Astrocytes	GFAP (1:50)		Goat	sc-6170, Santa Cruz
				Biotechnology
				
Isotype/serum controls:	Un-conjugated	IgG2b	Rat	IG-851125, Biosite
	Un-conjugated	IgG2a	Rat	P54605M, Biosite
	Un-conjugated	Serum	Goat	X0907, DakoCytomation
	Un-conjugated	Ig fraction	Rabbit	X0903, DakoCytomation
	Biotin-conjugated	IgG2a,κ	Mouse	553455, BD Biosciences
				
Secondary antibodies:				
Anti-rabbit IgG	Alexa Fluor594-conjugated (1:200)		Donkey	A21207, Invitrogen
Anti-rabbit IgG	Alexa Fluor546-conjugated (1:200)		Goat	A11010, Invitrogen
Anti-rabbit IgG	Alexa Fluor488-conjugated (1:200)		Goat	A11070, Invitrogen
Anti-rabbit IgG	Alexa Fluor488-conjugated (1:200)		Chicken	A21441, Invitrogen
Anti-rat IgG	Alexa Fluor568-conjugated (1:200)		Goat	A11077, Invitrogen
Anti-rat IgG	Alexa Fluor594-conjugated (1:200)		Goat	A11007, Invitrogen
Anti-rat Ig	Alexa Fluor488-conjugated (1:200)		Goat	A11006, Invitrogen
Streptavidin	HRP-conjugated Streptavidin (1:200)			P0397, DakoCytomation

PE: phycoerythrin, PerCP-Cy5.5: perinidin chlorophyll protein-cyanine5.5, APC: allophycocyanin, FITC: fluorescein isothiocyanate, vWF: von Willebrandt's Factor, PECAM-1: platelet endothelial adhesion molecule-1, GFAP: glial fibrillary acidic protein, GFP: green fluorescent protein.

#### Immunohistochemistry for GFP^+ ^cells

To detect infiltrating GFP^+ ^cells, sections were rinsed 3 × 15 min in 0.05 M Tris-buffered saline (TBS, pH 7.4), and endogenous peroxidase activity was blocked by rinsing the sections in 30% H_2_O_2_, methanol and TBS (1:1:8) for 30 min at room temperature (RT). After rinsing 2 × 10 min in TBS + 0.5% Triton, the sections were incubated with 10% fetal calf serum (FCS) in TBS for 30 min at RT to reduce non-specific staining. Sections were then incubated with rabbit anti-GFP (Table 1, Abcam) diluted 1:1,000 in TBS containing 10% FCS overnight at 4°C. Next, sections were rinsed 3 × 15 min in TBS + 0.5% Triton and incubated with peroxidase-labeled "ready-to-use" EnVision^+ ^polymer (DakoCytomation) overnight at 4°C. Sections were then rinsed 3 × 15 min in TBS and developed for 7 min in TBS containing 0.05% diaminobenzidine and 0.033% H_2_O_2_. Finally, sections were rinsed in TBS, dehydrated in a graded series of alcohol, cleared in xylene and mounted in Depex. Antibody specificity was assessed by negative controls using substitution of the primary antibody with rabbit IgG (Table 1, DakoCytomation) or omission of the primary antibody. These sections were devoid of signal.

#### Double immunofluorescence staining for cell specific markers and IL-1β and/or TNF-α

Sections were air dried, rinsed in TBS for 10 min, and blocked with 10% FCS in TBS + 0.5% Triton for 30 min at RT. Next, the sections were incubated with primary antibody (Table 1) overnight at 4°C, followed by rinsing for 10 min in TBS and incubation with a species-specific secondary fluorescent antibody (Table 1) diluted 1:200 for 1–2 hours the next day. From this point forward, the sections were protected from light. After 10 min in TBS, sections were incubated for 2 hours with the second primary antibody diluted in TBS + 0.5% Triton containing 10% FCS, rinsed in TBS for 10 min at RT, and then incubated with the secondary antibody diluted 1:200 in TBS + 0.5% Triton containing 10% FCS for 1–2 hours at room temperature (RT). Finally, sections were rinsed 2 × 10 min in TBS, followed by 2 × 10 min in distilled H_2_O, and mounted in ProLong^® ^Gold antifade reagent with DAPI (Invitrogen, Taastrup, Denmark). Negative controls, where primary antibodies were replaced by isotype controls (Table 1) or omitted from the protocol, were devoid of signal.

#### Estimation of numbers of infiltrating GFP^+ ^cells

The number of GFP^+ ^cells in the ipsilateral cortex was estimated using the cell counting method described in Lambertsen *et al*. [25]. GFP^+ ^cells with an identifiable nucleus were counted in approximately 16 sections from each animal, 384 μm apart. This was done using a 100× objective and a 25% frame area, stepping 300 μm/300 μm in the x-y position using the CAST Grid System from Olympus. The total number (N) of GFP^+ ^cells in each animal was estimated using the formula: Estimate of N = ΣQ × (1/ssf) × (1/asf) × (1/tsf), where Q was the number of cells counted, 1/ssf the sampling section fraction (1/ssf = 24), and 1/asf the area sampling fraction (1/asf = 36.4) [26]. The thickness sampling fraction (1/tsf) was set to 1, since cells were counted in the entire height of the sections [25].

### Flow cytometry

#### Preparation of cell suspensions

Tissues from individual mice were processed separately. Single-cell suspensions of ipsi- or contralateral cortices or lymph nodes were obtained by homogenisation using 70-μm nylon cell strainers (BD Falcon, Franklin Lakes, NJ, USA) [24,27], in RPMI containing 10% FCS and 1 μl/ml BD GolgiPlug™ with Brefeldin A (BD Biosciences). Cells were transferred to sterile petri dishes and placed in a 37°C incubator with 5% CO_2 _for 4 1/2 hours. Cell suspensions were transferred to polystyrene tubes and pelleted by centrifugation (1,400 rpm for 6 min at 4°C).

#### Staining procedures

Cells were incubated for 30 min in staining-buffer (staining-buffer: Hank's Buffered Salt Solution with 2% FCS and 0.1% sodium azide), containing 50 μg/ml Syrian hamster Ig (Jackson Immunoresearch, West Grove, PA, USA) and 1 μg/ml anti-FcγIII/II receptor (BD FcBlock™, BD Biosciences), to block non-specific staining. After rinsing, cells were stained for surface antigens (or their isotype controls) (Table 1) for 30 min at RT, and then rinsed in staining-buffer [24,27]. Following staining of surface markers (CD11b, CD45, Gr1, TCRβ), the cells were fixed and permeabilized in Cytofix/Cytoperm™ (BD Biosciences) for 20 min at 4°C, washed in 1 ml 1× PermWash™ buffer (BD Biosciences), and incubated with antibodies recognizing intracellular antigens (IL-1β, TNF-α or isotype controls) (Table 1) for 30 min at RT. Cells stained with unconjugated antibodies were rinsed, then incubated with fluorescently labelled goat anti-rabbit secondary antibodies for 30 min at RT. After the final rinse, cells were resuspended in staining-buffer.

#### Blood

For blood samples, cells were stained using antibodies recognizing CD11b, CD45 and TCRβ (Table 1), as described for brain homogenates. Red blood cells were then lysed using Serotec Erythrolyse Red Blood Cell buffer (Serotec) diluted 1:10 in distilled water for 7 min at RT. Finally, cells were rinsed three times and resuspended in staining-buffer.

#### Flow cytometric analysis

Staining was analyzed using a FACSCalibur flow cytometer and CellQuest Pro Software (BD Biosciences). Estimation of numbers of cells was done as previously described [24,27]. Data are presented as an average of these numbers. Preparation and staining of cortical homogenates inevitably resulted in cell loss, and the cell numbers and proportions presented are based on cells remaining in suspension. For analysis of microglia and macrophages/granulocytes, cells were gated on side scatter (SSC) versus CD11b, followed by forward scatter (FSC) versus CD11b, and FSC versus CD45. Microglia were separated from macrophages/granulocytes based on CD45 expression, with intermediate levels of CD45 (CD45^dim^) identifying CD11b^+ ^parenchymal microglia, and high levels of CD45 (CD45^high^) identifying CD11b^+ ^macrophage/granulocytes [28]. GFP^+^, Gr1^+^, IL-1β^+ ^and TNF-α^+ ^cells were identified on additional colour channels. GFP^+ ^BM-derived cells were separated from GFP^- ^resident cells based on GFP expression. For quantification of IL-1β/TNF-α co-expression, resident microglia (GFP^-^CD45^dim^) and infiltrating leukocytes (GFP^+^CD45^high^) were gated on SSC versus CD45 followed by FSC versus CD45. For analysis of expression of IL-1β and TNF-α in TCRβ^+ ^T cells, cells were gated on SSC versus CD45 followed by FSC versus CD45. IL-1β^+^, TNF-α^+ ^and TCRβ^+ ^cells were identified on additional colour channels. Positive staining was determined based on fluorescence levels of isotype controls or autofluorescence controls (for GFP).

### Data analysis

Histology results were documented using an Olympus DP70 digital camera mounted on an Olympus BX51 microscope connected to a PC with Olympus DP-software. Figures were organized using Adobe Photoshop CS. The data were evaluated using Kruskal-Wallis test (non-parametric ANOVA) with Dunn's multiple comparison test as follow-up test. Comparisons of mean values between two groups of mice were done using the non-parametric Mann-Whitney test using the GraphPad Prism 4.0b software for Macintosh, GraphPad Software, San Diego, California, USA. Data are presented as means ± SD. Statistically significant differences were established at P < 0.05.

## Results

### Regional distribution of cells producing IL-1β and TNF-α

**Figure 1:**
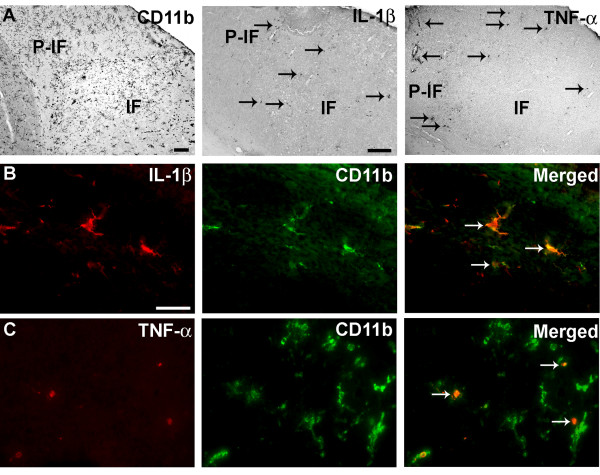
**Co-expression of IL-1β or TNF-α with CD11b^+ ^cells**. Cortical infarction 24 hours after pMCAO leads to expression of IL-1β and TNF-α in activated CD11b^+ ^microglia and macrophages/granulocytes. (A) Distribution of CD11b^+^, IL-1β^+ ^and TNF-α^+ ^cells in infarct (IF) and peri-infarct (P-IF) regions. IL-1β^+ ^(B) and TNF-α^+ ^(C) cells were particularly numerous at the edge of the infarct, and these cytokines were exclusively expressed by CD11b^+ ^cells (arrows). CD11b^+ ^cells were visualized using Alexa Fluor^® ^488-conjugated goat anti-rat IgG, IL-1β^+ ^and TNF-α^+ ^cells using Alexa Fluor^® ^594-conjugated donkey anti-rabbit IgG. Scale bars: 200 μm (A), 20 μm (B, C).

We have previously shown that IL-1β and TNF-α are produced by CD11b^+ ^microglia and infiltrating CD11b^+ ^cells, i.e. macrophages and granulocytes, situated within and at the edge of the infarct 24 hours after pMCAO in SJL and C57BL/6 mice [12,14,20]. In line with these observations, IL-1β- and TNF-α-expressing cells were observed within and adjacent to the infarct in B6.SJL-Ptrpr^a ^Pepc^b^/BoyJ mice 24 hours after pMCAO. These cells co-localized with activated CD11b^+ ^microglia and CD11b^+ ^macrophages/granulocytes observed in parallel sections (Figure 1A). Double immunofluorescence staining confirmed that IL-1β (Figure 1B) and TNF-α Figure 1C) were expressed by activated CD11b^+ ^microglia and infiltrating CD11b^+ ^macrophages/granulocytes.

### Blood-borne, bone marrow-derived cells infiltrate after pMCAO

**Figure 2:**
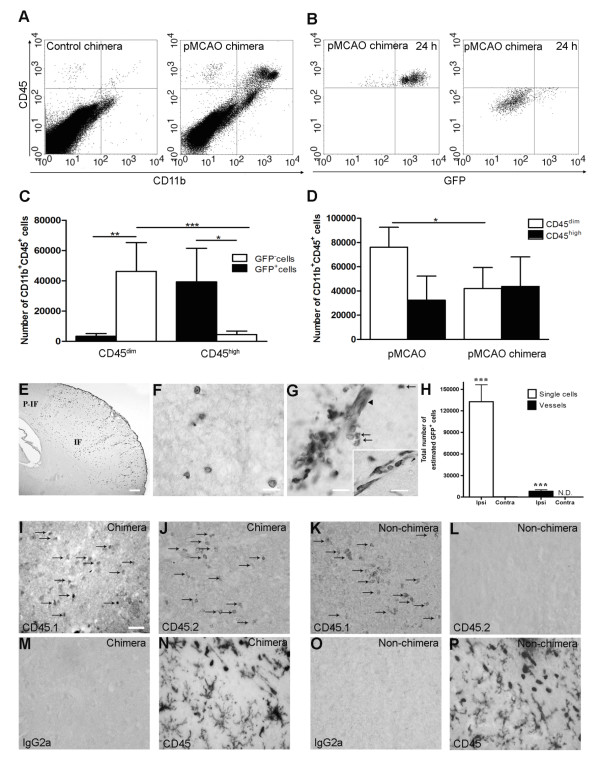
**Infiltration of GFP^+ ^BM-cells in infarct and peri-infarct regions**. (A-B) Dot plots of viable macrophages/granulocytes (CD11b^+^CD45^high^, top right quadrants) and microglia (CD11b^+^CD45^dim^, bottom right quadrants) in cortex from BM-chimeric unmanipulated mice and mice exposed to pMCAO. (C) Bar graph showing mean numbers of CD11b^+^CD45^dim ^microglia and CD11b^+^CD45^high ^macrophages/granulocytes in BM-chimeric mice 24 hours after pMCAO, subdivided based on expression of GFP (n = 5). Approximately 92% of of the CD45^high ^population were GFP^+ ^. (D) Estimation and comparison of mean numbers of CD11b^+^CD45^dim ^microglia in non-chimeric (n = 10) versus BM-chimeric mice (n = 5) 24 hours after of pMCAO shows significantly fewer CD11b^+^CD45^dim ^microglial cells in irradiated mice. (E) Overview, showing distribution of infiltrating GFP^+ ^BM-derived cells into infarct (IF) and peri-infarct (P-IF) regions 24 hours after pMCAO. (E-G) By 24 hours, GFP^+ ^single cells (F) and vessel-associated aggregates of GFP^+ ^cells (arrows in G) were observed in infarct and peri-infarct regions. Some of the vessel-associated cells were round, leukocyte-like cells (arrows) while others were elongated cells lining the vasculature (arrow heads in G and in insert). (H) Bar graph showing mean numbers of single GFP^+ ^cells and vessel-associated aggregates of GFP^+ ^cells in ipsi- and contralateral cortex 24 hours after surgery (n = 10). (I-P) Immunohistochemical staining of CD45.1 (I, K), CD45.2 (J, L), IgG2a (M, O) and CD45 (N, P) in ischemic tissue in BM-chimeric (I, J, M, N) and non-chimeric mice (K, L, O, P) 24 hours after pMCAO. N.D, none detected. Scale bars: 200 μm (A), 10 μm (B, C). 50 μm (I-P) *P < 0.05, **P < 0.01, and ***P < 0.001.

Whole-body irradiated mice were reconstituted with BM cells from GFP-Tg mice, allowing us to distinguish infiltrating GFP^+^CD11b^+^CD45^high ^BM-derived cells from resident GFP^-^CD11b^+^CD45^dim ^microglia. Flow cytometric analysis showed that 97% of nucleated blood cells were GFP^+^, providing us with a reliable tool to differentiate between infiltrating GFP^+ ^BM-derived cells and resident GFP^- ^cells. Flow cytometric analysis of perfused cortex from chimeric mice allowed to survive 24 hours after pMCAO showed a substantial increase in CD11b^+^CD45^high ^cell population compared to unmanipulated control chimeras (Figure 2A). As expected, the majority of CD45^high ^cells were GFP^+ ^(92%), consistent with these cells being BM-derived (Figure 2B). Investigation of the CD11b^+^CD45^dim ^microglial population showed that these cells were primarily GFP^-^. However, there was evidence of injury-induced recruitment of GFP^+ ^microglial precursors (7%) into the ischemic cortex (Figure 2B, C). Comparison of the CD11b^+^CD45^high ^cell population in BM-chimeric mice and non-chimeric mice allowed to survive 24 hours after pMCAO (Figure 2D) showed similar mean numbers of CD11b^+^CD45^high ^infiltrating cells. In contrast, however, the CD11b^+^CD45^dim ^microglial population was significantly reduced in BM-chimeric compared to non-chimeric mice (Figure 2D) suggesting that whole body irradiation affects the resident microglial population, independent of injury. Similarly, the result showed less CD11b^+^CD45^dim ^microglia in chimeric versus unmanipulated non-chimeric mice (Figure 2A and 4A).

The distribution of infiltrating GFP^+ ^cells was visualized using immunohistochemistry. Twenty-four hours after pMCAO, GFP^+ ^cells were distributed throughout the infarct, such that the border of the infarct was easily delineated (Figure 2E). The GFP^+ ^cells occurred both as single cells (Figure 2F), and as vessel-associated aggregates (Figure 2G). Microscopic analysis of the perivascular vessel-associated aggregates showed two morphologically different cell types; round cells, which appeared to locate to the juxtavascular space and to infiltrate the neuropil (Figure 2G), and elongated cells lining the microvascular wall (insert in Figure 2G). In order to determine the extent of cellular recruitment, we estimated the total number of GFP^+ ^cells within the ischemic cortex using both morphometry and flow cytometry. By morphometry, the total number of single GFP^+ ^cells was estimated to be 130,000 ± 24,000 (mean ± SD) cells (Figure 2H). The number of vessel-associated aggregates of GFP^+ ^cells were quantified separately (8,000 ± 2,200) (Figure 2H). Only a few GFP^+ ^single cells were present in the cortex of the contralateral hemisphere (Figure 2H), or in brains from unmanipulated BM-chimeric mice. GFP^+ ^cells were identified in sections from all organs from BM-chimeric mice (data not shown). Using flow cytometry, we estimated the total number of GFP^+ ^cells within the isolated cortex to be 42,655 ± 23,465 (mean ± SD) cells (Figure 2C), which corresponded to 33% of the number of GFP^+ ^cells estimated by morphometry (Figure 2H).

**Figure 3:**
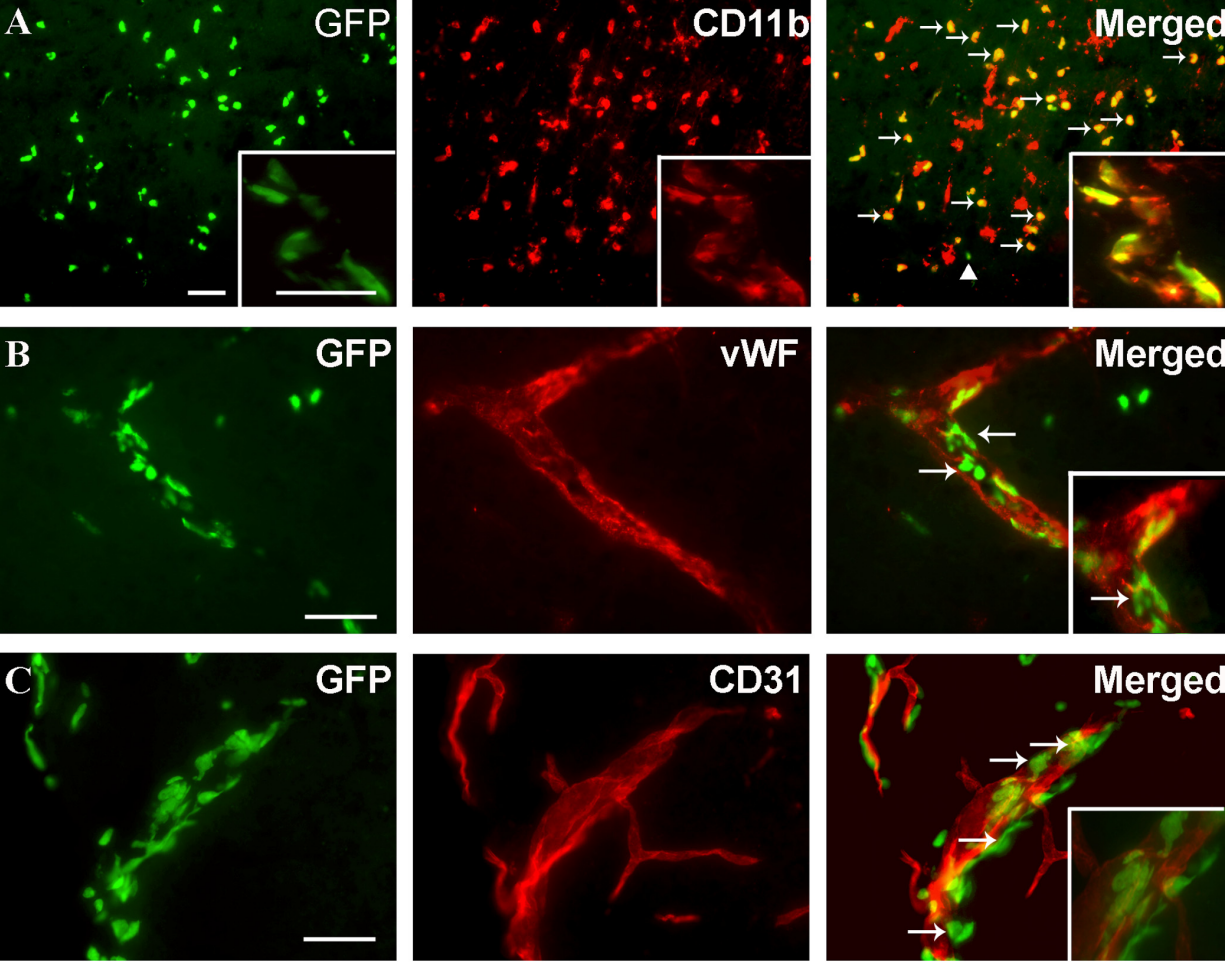
**BM-derived GFP^+ ^single cells and vessel-associated cells express CD11b**. Fluorescence microscopy for GFP combined with immunofluorescence detection of (A) CD11b, (B) vWF and (C) CD31, 24 hours after pMCAO. (A) Fluorescence detection of GFP and CD11b showed that most GFP^+ ^cells co-expressed CD11b (yellow cells, indicated by arrows), and intermingled with CD11b^+ ^host cells. Note also that a few GFP^+ ^cells did not co-express CD11b (arrow head). Insert shows high magnification of GFP^+ ^cells, some of which co-express CD11b, aggregated around a vessel. (B, C) Fluorescence detection of GFP and the endothelial cell markers vWF (B) and CD31 (C). Inserts show higher magnification of sections of the same vessels. Although there are indications that single vWF^+ ^cells co-express GFP (arrows in B), this could not be reproduced using staining for CD31, and the majority of vWF^+ ^and CD31^+ ^cells showed no co-expression of GFP. Instead, GFP remained confined to round and elongated cells located in the juxtavascular space (insert in C). CD11b^+ ^cells were visualized using Alexa Fluor^® ^568-conjugated goat anti-rat IgG, vWF^+ ^and CD31^+ ^cells using Alexa Fluor^® ^546-conjugated goat anti-rabbit IgG and Alexa Fluor^® ^594-conjugated goat anti-rat IgG, respectively. Scale bars: 20 μm (A-C).

Because we observed a reduced number of CD11b^+^CD45^dim ^microglia after pMCAO in BM-chimeric mice versus non-chimeric mice (Figure 2D), we investigated whether irradiation affected cellular CD45 expression resulting in incorrect estimation of cell numbers. Immunohistochemical stainings for CD45.1 (Figure 2I, K) and CD45.2 (Figure 2J, L) confirmed the presence of donor-derived CD45.2^+ ^cells within the ischemic cortex of BM-chimeric mice (Figure 2J) but not in non-chimeric mice (Figure 2L). Similarly, we found no evidence of altered CD45 expression when comparing stainings with a generally expressed CD45 marker in chimeric and non-chimeric mice 24 hours after pMCAO (Figure 2N, P). Fluorescence microscopy confirmed that the majority of GFP^+ ^cells were CD11b^+ ^(Figure 3A), thereby showing that they were myeloid lineage-derived macrophages/granulocytes. This was additionally emphasized by the staining for the endothelial markers von Willebrand Factor (vWF) and platelet endothelial cell adhesion molecule-1 (PECAM or CD31), which appeared to be expressed largely by GFP^- ^cells (Figure 3B, C).

### Kinetics of the microglial and macrophage/granulocyte response

**Figure 4:**
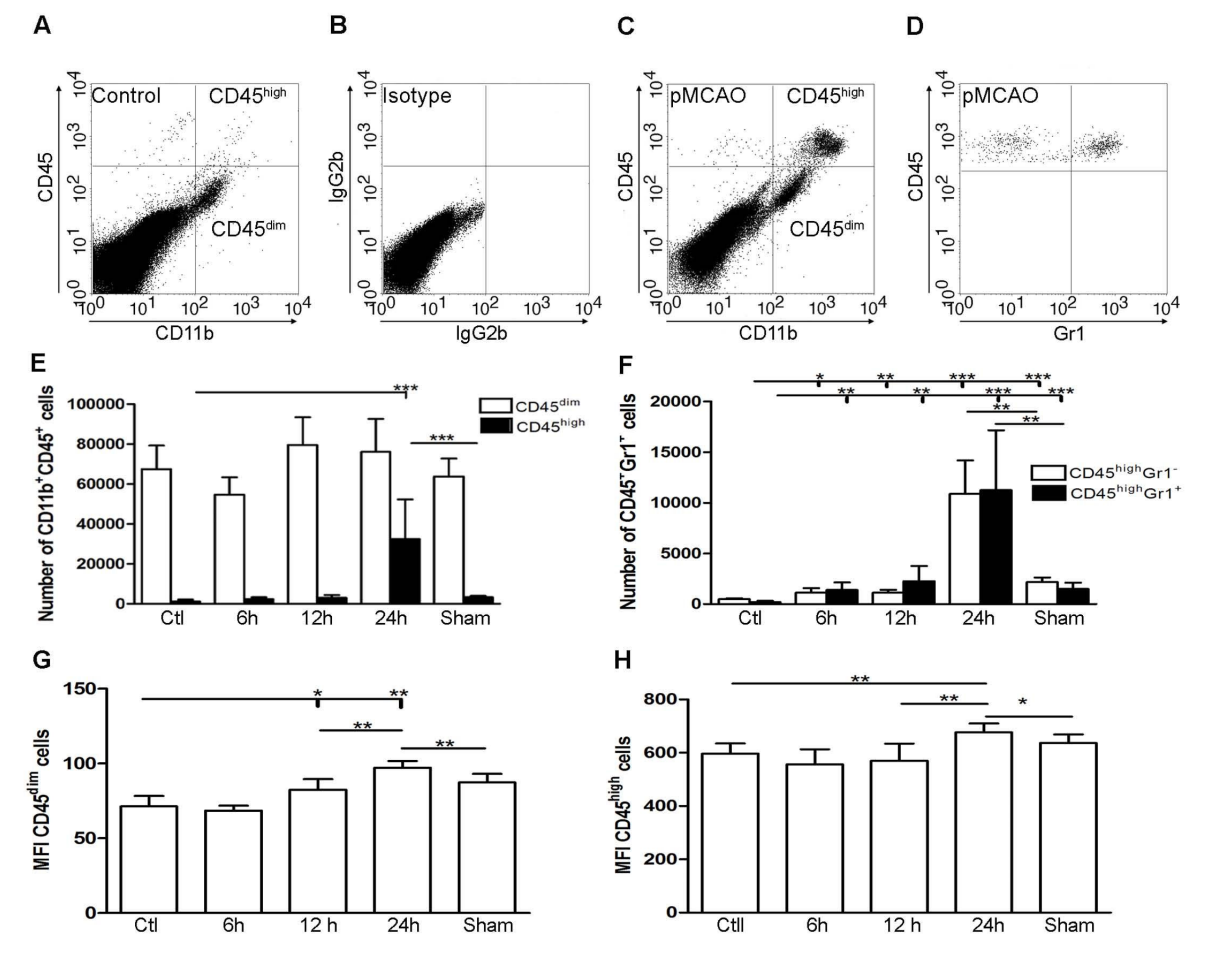
**Inflammatory response following permanent MCA occlusion**. (A-C) Dot plots of viable CD11b^+^CD45^high ^macrophages/granulocytes (top right quadrants) and CD11b^+^CD45^dim ^microglia (bottom right quadrants) in cortex from unmanipulated control mice (A, B), and mice exposed to pMCAO with 24 hour survival (C). (D) At 24 hours, flow cytometric analysis of the CD11b^+^CD45^high ^profiles showed that approximately half of the population consisted of CD45^high^Gr1^+ ^granulocytes. (E) Quantification of CD11b^+^CD45^dim ^and CD11b^+^CD45^high ^cells in unmanipulated control mice (n = 10), in mice 6 (n = 7), 12 (n = 7), or 24 hours after pMCAO (n = 10), and in sham-operated mice 24 hours after pMCAO (n = 7). (F) Bar graphs showing equal recruitment of CD11b^+^CD45^high^Gr1^- ^macrophages and CD11b^-^CD45^high^Gr1^+ ^granulocytes in unmanipulated mice, in mice 6, 12, or 24 hours after pMCAO, and in sham-operated mice 24 hours after pMCAO. (G, H) Bar graphs showing the mean fluorescent intensity (MFI) of CD45 expression by CD45^dim ^microglia (G) and CD45^high ^macrophages/granulocytes (H). *P < 0.05, **P < 0.01, and ***P < 0.001.

Having shown that the vast majority of the GFP^+^CD11b^+^CD45^+^cells were infiltrating BM-derived macrophages/granulocytes, and that almost all CD11b^+^CD45^dim ^cells were resident microglia, we investigated the kinetics of microglial activation and cell infiltration after pMCAO in non-chimeric mice, using CD45 levels to distinguish resident CD45^dim ^microglia from infiltrating CD45^high ^macrophages/granulocytes by flow cytometry (Figure 4A–D). Estimation of the numbers of CD11b^+^CD45^dim ^microglia revealed comparable cell numbers in brains of unmanipulated mice and mice after 6 hours of survival, whereas there was a significant increase in microglia 12 hours or 24 hours after pMCAO (Figure 4E). As expected, the numbers of microglia in sham-operated mice remained unaffected at 24 hours (Figure 4E). Furthermore, CD11b^+^CD45^high ^macrophages/granulocytes increased in numbers over the period from 6 to 24 hours post-occlusion, compared to unmanipulated controls and sham-operated mice at 24 hours (Figure 4E). Two populations of CD11b^+^CD45^high ^cells were visible on flow cytometric dot plots from mice exposed to 24 hours of pMCAO, which differed by expressing intermediate or high levels of CD11b (Figure 4C). Since CD11b is expressed both on macrophages and granulocytes [21,22], we investigated whether and to what extent CD45^+^Gr1^+ ^granulocytes contributed to the CD11b^+^CD45^high ^cell population (Figure 4D, F). There was significant infiltration by granulocytes as early as 6 hours compared to unmanipulated control mice (Figure 4F). By 24 hours, there was an additional pMCAO-induced increase in CD45^+^Gr1^+ ^cells compared to sham-operated mice with 24 hours survival. Similar numbers of CD45^+^Gr1^+^granulocytes and CD45^+^Gr1^- ^macrophages were recruited to the ischemically injured cortex, and these populations shared the same temporal profile (Figure 4F).

Finally, since microglia become activated by stroke [14,20], and evidence shows that CD45 is inducible in microglia [29], we investigated whether cellular levels of CD45 expression were affected by the ischemic insult. For this, we analysed mean fluorescence intensity (MFI) of the CD45 signal on CD11b^+^CD45^dim ^microglia and CD11b^+^CD45^high ^macrophages/granulocytes. MFI values were obtained for unmanipulated controls, in mice with 6-, 12-, and 24-hour survivals, and in sham-operated mice at 24 hours. We observed a significant upregulation of CD45 on microglia by 12 hours, which reached peak levels 24 hours after pMCAO compared to unmanipulated controls and sham-operated mice (Figure 4G). Similarly, CD45 levels were increased on infiltrating macrophages/granulocytes by 24 hours compared to unmanipulated control and sham-operated mice (Figure 4H).

### IL-1β and TNF-α are expressed by largely non-overlapping subsets of cells

**Figure 5:**
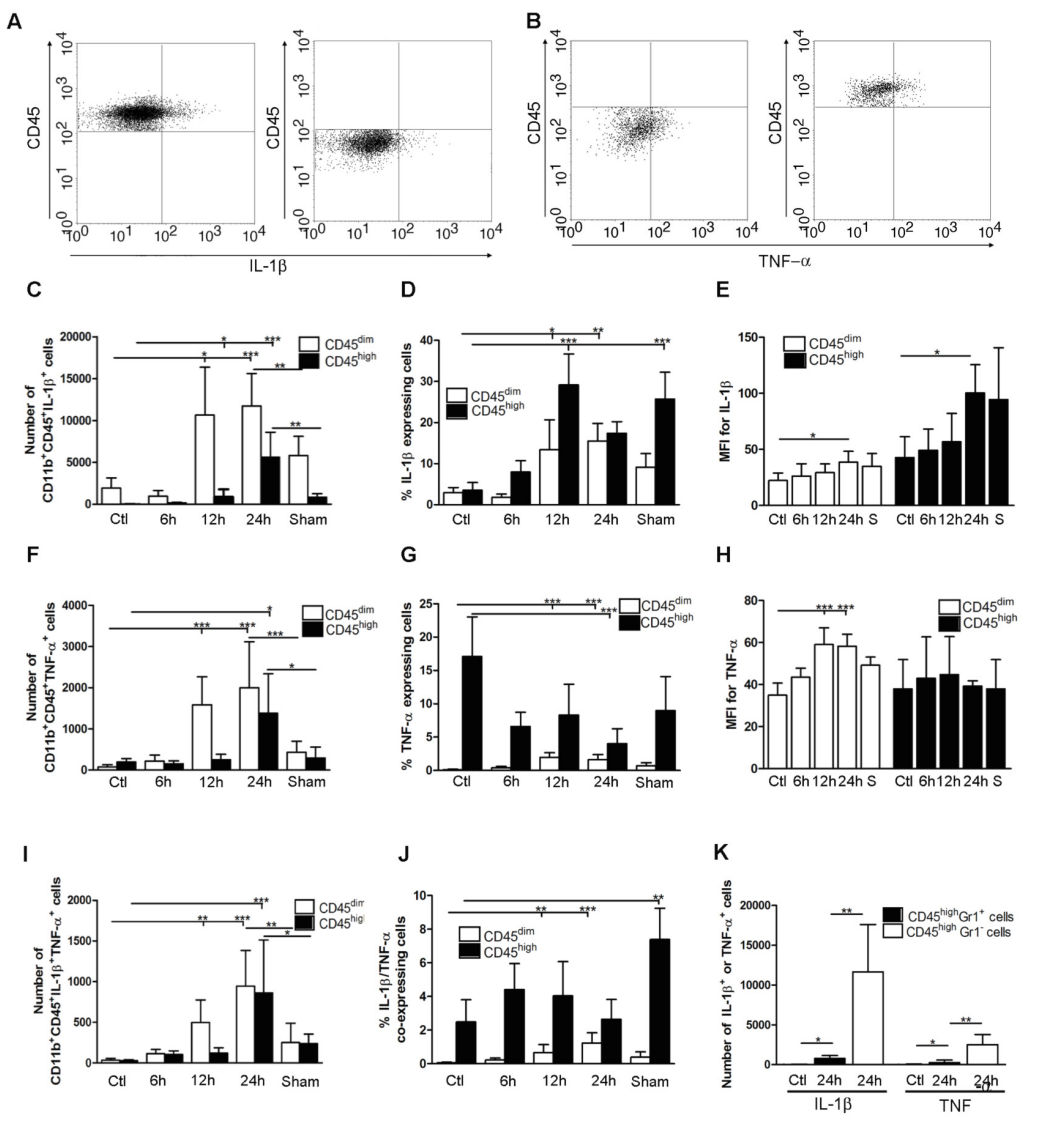
**Cytokine expression in segregated populations of cells following stroke**. (A, B) Dot plots showing CD11b^+^CD45^high ^macrophages/granulocytes (upper right quadrants) and CD11b^+^CD45^dim ^microglia (bottom right quadrants) expressing IL-1β (A) or TNF-α (B). (C-J) Bar graphs showing numbers and proportions of IL-1β (C, D), TNF-α (F, G) and IL-1β/TNF-α co-expressing (I, J) CD11b^+^CD45^dim ^microglia and CD11b^+^CD45^high ^macrophages/granulocytes in unmanipulated control mice (n = 10), in mice 6 (n = 7), 12 (n = 7), or 24 hours after pMCAO (n = 10), and in sham-operated mice 24 hours after pMCAO (n = 7). (E, H) Comparison of the MFI values for IL-1β (E) and TNF-α (H) in viable CD11b^+^CD45^dim ^microglia and CD11b^+^CD45^high ^macrophages/granulocytes in unmanipulated mice, in mice 6, 12, or 24 hours after pMCAO, and in sham-operated mice 24 hours after pMCAO. Macrophages/granulocytes express significantly more IL-1β than do microglial in unmanipulated mice, in mice 6, 12, or 24 hours after pMCAO, and in sham-operated mice 24 hours after pMCAO (E), whereas microglial cells express significantly higher levels of TNF-α than do macrophages/granulocytes at 12 h and 24 hours, and in sham-operated mice 24 hours after pMCAO (H). (K) CD11b^+^CD45^high^Gr1^- ^macrophages and not CD11b^+^CD45^high^Gr1^+ ^granulocytes are the main producers of IL-1β and TNF-α 24 hours after pMCAO. *P < 0.05, **P < 0.01, and ***P < 0.001.

Next we used flow cytometry to determine which cell population(s), the resident CD11b^+^CD45^dim ^microglia or the infiltrating CD11b^+^CD45^high ^macrophages/granulocytes, or both, expressed IL-1β, TNF-α, or a combination of IL-1β and TNF-α after pMCAO (Figure 5A, B). Numbers of IL-1β-expressing CD11b^+^CD45^dim ^microglia and infiltrating CD11b^+^CD45^high ^macrophages/granulocytes peaked between 12 and 24 hours after pMCAO (Figure 5C). The proportion of IL-1β-expressing CD11b^+^CD45^dim ^microglia increased from 3% of the total microglial population in unmanipulated mice to 15% of the total microglial population 24 hours after pMCAO (Figure 5D). In addition, the proportion of IL-1β-expressing CD11b^+^CD45^high ^macrophages/granulocytes increased from 3% in unmanipulated mice to 29% by 12 hours, then decreased to 17% of the total macrophage/granulocyte population after 24 hours of pMCAO (Figure 5D). MFI values for IL-1β showed increased protein expression by CD11b^+^CD45^dim ^microglia and infiltrating CD11b^+^CD45^high ^macrophages/granulocytes after 24 hours of pMCAO (Figure 5E).

The time profile for TNF-α-expressing CD11b^+^CD45^dim ^microglia showed a significant increase in numbers of TNF-α^+ ^cells at 12 hours, which peaked 24 hours after pMCAO compared to unmanipulated control mice. Similarly there was a injury-induced increase in numbers of CD11b^+^CD45^high ^macrophages/granulocytes that expressed TNF-α at 24 hours after pMCAO (Figure 5F). Calculation of the proportion of TNF-α-expressing CD11b^+^CD45^dim ^microglia and CD11b^+^CD45^high ^macrophages/granulocytes showed that the proportion of TNF-α^+ ^microglia peaked with 2% of the CD11b^+^CD45^high ^population at 12–24 hours after pMCAO, while the proportion of TNF-α^+ ^macrophages/granulocytes was 17% of the CD11b^+^CD45^high ^population in unmanipulated controls and 4% at 24 hours after pMCAO. This most of all reflected that a relatively large proportion of the very few CD11b^+^CD45^high ^cells present in the unmanipulated mouse cortex expressed TNF-α (Figure 5G). Generation of MFI values for TNF-α showed a significant increase in the amount of TNF-α expressed by microglia by 12 and 24 hours compared to unmanipulated control mice. In contrast, the level of TNF-α expression by CD11b^+^CD45^high ^macrophages/granulocytes seemed unaffected by ischemic injury (Figure 5H).

We additionally identified a subpopulation of CD11b^+^CD45^dim ^microglia and CD11b^+^CD45^high ^macrophages/granulocytes that co-expressed IL-1β and TNF-α (Figure 5I). The proportion of IL-1β/TNF-α co-expressing microglia increased from 0.05% in unmanipulated controls to 1.2% after 24 hours of pMCAO, whereas co-expressing macrophages doubled from 2% in unmanipulated controls to 4% after 12 hours of pMCAO, before declining to 2% after 24 hours (Figure 5J).

Interestingly, the proportions of IL-1β-, TNF-α-, and IL-1β/TNF-α-expressing macrophages/granulocytes were relatively high in sham-operated mice (Figure 5D, G, J). However, since the number of CD11b^+^CD45dim microglia and CD11b^+^CD45high macrophages/granulocytes did not differ between unmanipulated controls and sham-operated mice (Figure 4E), the overall number of cytokine expressing cells was much lower in sham-operated versus pMCAO operated mice (Figure 5C, F, I).

Taken together, these results clearly show that largely non-overlapping subsets of microglia and macrophages/granulocytes produce IL-1β and TNF-α after pMCAO in mice.

Finally, since both macrophages and granulocytes express CD11b and high levels of CD45 [21], we used Gr1 to determine which cell population was the predominant CD45^high ^source of IL-1β and TNF-α. We found a small but significant increase in both IL-1β- and TNF-α expressing CD45^high^Gr1^+ ^granulocytes at 24 hours after pMCAO compared to unmanipulated control mice. However, the CD45^high^Gr1^- ^macrophages were clearly the major producers of both IL-1β and TNF-α (Figure 5K). A small number of CD45^high ^cells identified as T cells (TCRβ^+^CD45^high^) was also observed. The number of T cells in ischemic cortex by 6, 12 and 24 hours did not differ from the number in cortex of unmanipulated or sham-operated mice (< 200), and none of the TCRβ^+^CD45^+ ^T cells were observed to express either IL-1β or TNF-α (data not shown).

### Comparison of cytokine expression in LPS-stimulated and ischemia-activated macrophages

**Figure 6:**
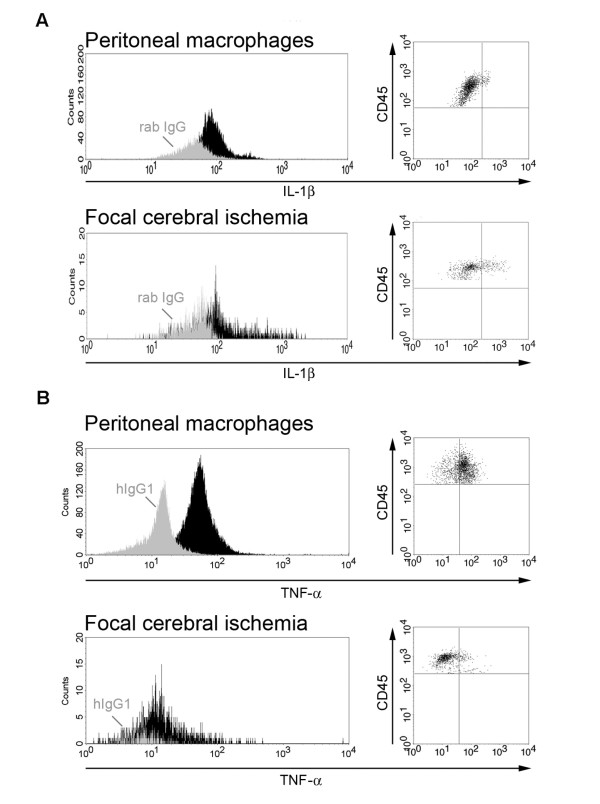
**Sensitivity of cytokine detection using flow cytometry**. Histograms and dot plots of IL-1β (A) and TNF-α (B) expression in LPS-activated peritoneal macrophages versus macrophages/granulocytes isolated from cortex 24 hours after pMCAO. Light colored histograms represent cells stained with isotype control antibodies and filled histograms represent cells stained with antibodies for either IL-1β (A) or TNF-α (B).

To further verify our findings, we compared the intensity of the fluorescent cytokine signal between macrophages obtained from mice after 24 hours of pMCAO and LPS-stimulated, thioglycolate-elicited peritoneal macrophages, used as a positive control for IL-1β and TNF-α expression. Histograms shown in Figure 6 demonstrate a clear shift from the respective control antibody (IgG) for both IL-1β (Figure 6A) and TNF-α (Figure 6B). Determination of the MFI showed that the LPS-stimulated peritoneal macrophages expressed lower levels of IL-1β (MFI: 85.28, (IgG: 49.23)) than did ischemia-activated macrophages (MFI: 104.03 (IgG: 46.06)) whereas LPS-stimulated peritoneal macrophages expressed higher levels of TNF-a (MFI: 53.84, (IgG: 14.79)) than did ischemia-activated macrophages (MFI: 39.28 (IgG: 24.11)).

### Expression of IL-1β and TNF-α by Gr1^-^CD11b^+ ^microglia and macrophages

**Figure 7:**
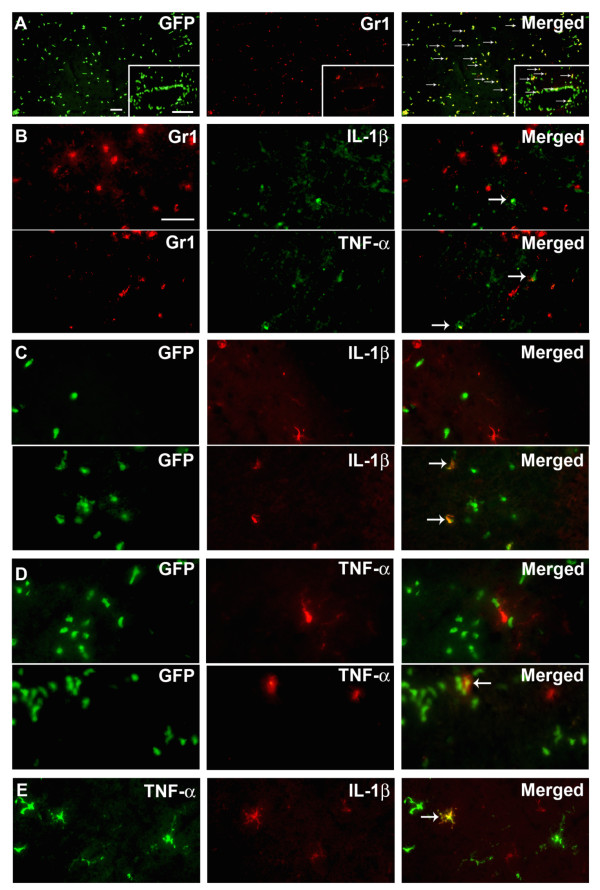
**IL-1β and TNF-α are expressed by macrophages and microglia**. (A) Infiltrating GFP^+ ^cells consist of Gr1^+ ^granulocytes and Gr1^- ^macrophages. (B) Double immunofluorescence for Gr1^+ ^granulocytes and either IL-1β or TNF-α showed no detectable co-expression 24 hours after pMCAO. Immunofluorescence detection of GFP and IL-1β (C) or TNF-α (D) showed that  these cytokines are expressed by resident GFP^- ^microglia and infiltrating GFP^+ ^macrophages (E) Immunofluorescence double staining confirmed the flow cytometry results by showing that IL-1β and TNF-α are expressed by largely segregated subpopulations of cells. Very few IL-1β^+^TNF-α^+ ^co-expressing cells were identified during microscopic analysis (E). Gr1^+ ^granulocytes were visualized using Alexa Fluor^® ^594-conjugated goat anti-rat IgG, and IL-1β^+ ^and TNF-α^+ ^cells using both Alexa Fluor^® ^488-conjugated goat anti-rabbit and chicken anti-rabbit IgG. Scale bars: 50 μm (A) 20 μm (insert, A), 20 μm (B-E).

The flow cytometry data presented above show that microglia and macrophages, but not granulocytes, are major producers of IL-1β and TNF-α, and that co-expression of IL-1β and TNF-α is limited to a small subset of microglia and macrophages. We then validated these results using double-immunofluorescence of tissue sections, to distinguish resident microglia from infiltrating cells in BM-chimeric mice. Because a subpopulation of the infiltrating GFP^+ ^cells were identified as granulocytes (Figure 7A), we first investigated if Gr1^+ ^granulocytes express IL-1β and TNF-α by using double immunofluorescence. We found that neither IL-1β nor TNF-α was expressed by Gr1^+ ^granulocytes (Figure 7B). This might reflect the lower sensitivity of immunohistochemistry compared to flow cytometry, or may indicate that granulocytes express smaller amounts of protein (IL-1β MFI: 58 ± 18; TNF-α MFI: 27 ± 2) than do macrophages (IL-1β MFI: 104 ± 25; TNF-α MFI: 40 ± 2) (P < 0.004). Double-immunofluorescence staining identified both resident and infiltrating cells expressing either IL-1β (Figure 7C) or TNF-α (Figure 7D), but only rarely identified cells co-expressing IL-1β and TNF-α 24 hours after pMCAO (Figure 7E), confirming the flow cytometric data. There was no co-localization of IL-1β or TNF-α staining with the astroglial marker GFAP (data not shown) or with the endothelial markers vWF/CD31 (data not shown).

## Discussion

We have shown for the first time that IL-1β and TNF-α are produced by largely non-overlapping subsets of microglia and macrophages after induction of ischemic stroke in mice. Using flow cytometry and histology, validated by the use of BM-chimeric mice, we showed that microglia and macrophages are the major producers of IL-1β and TNF-α after pMCAO, and that at maximum 1.2% of microglia and 4.5% of infiltrating macrophages co-express IL-1β and TNF-α within the first 24 hours after pMCAO. Granulocytes, which accounted for 50% of the CD11b^+^CD45^high ^cell population and which are known to exacerbate ischemic brain damage [30], accounted for only 2.4% of IL-1β and 0.8% of TNF-α expression by CD11b^+^CD45^high ^cells after 24 hours of pMCAO, indicating that the contribution of these cells to the production of IL-1β and TNF-α after pMCAO in mice is negligible. A scenario therefore emerges wherein different subsets of microglia and macrophages may have different roles in ischemic stroke, and may thus either improve or reduce the chance of survival of ischemic neurons.

Our observation of a peak in the total number of IL-1β- and TNF-α-expressing cells 24 hours after pMCAO is in line with previous demonstrations of a time-dependent peak in the number of IL-1β mRNA-, TNF-α mRNA-, and TNF-α protein-expressing cells in SJL and C57BL/6 mice, 12–24 hours after pMCAO [12,14,25]. By taking advantage of the ability of flow cytometry to distinguish between CD11b^+^CD45^dim ^microglia and infiltrating CD11b^+^CD45^high ^macrophages, we showed that the number of IL-1β- and TNF-α-expressing microglia by far exceeded the number of cytokine-expressing macrophages 12 hours after pMCAO. This result was expected based on the known steady increase in the number of infiltrating macrophages over the first 24 hours of pMCAO, but this result has not been previously demonstrated.

It was striking to observe that a relatively large proportion of the small number of macrophages present in the cortex from unmanipulated mice express TNF-α, and that the MFI levels of TNF-α expression in these cells are comparable to mean cellular TNF-α expression levels in pMCAO and sham-operated mice. This indicates that TNF-α expression by macrophages is relatively constant no matter whether the cells have infiltrated the cortex prior to or after pMCAO. Interestingly, the proportion of cytokine-expressing macrophages in sham-operated mice was at least as high as that in mice subjected to pMCAO. This likely reflects that sham surgery in itself induces a focal lesion in the cortex [12,14]. It is important to note, however, that overall numbers of cytokine-expressing macrophages are not increased versus control mice, since there was no significant recruitment of CD11b^+^CD45^high ^cells to the cortex of sham-operated mice.

Permanent MCAO results in formation of a pan-necrotic infarct, with loss of all cell types including microglial cells. Although neurons and microglia can still be clearly detected after 6 hours of pMCAO [12,14], the developing infarct is usually characterised by severe cell loss 12 hours after pMCAO [12,14,20] We were therefore surprised not to observe a reduction in the number of CD11b^+^CD45^dim ^microglia 12 and 24 hours after pMCAO. We wondered whether determination of upregulation of CD45, which is widely used to detect both resting and activated microglia in flow cytometry [24,27-29,31] and histology [32,33], might lead to increased detection. Indeed, MFI analysis showed that CD45 levels are upregulated in microglia 12–24 hours after pMCAO. However, microglial expression of CD45 was far below that expressed by macrophages/granulocytes, confirming results by others [34]. Using a bone marrow chimeric approach, we observed that approximately 7% of the CD45^dim ^microglia were GFP^+ ^after 24 hours of pMCAO, suggesting that BM-derived microglial precursors could also contribute to the expansion of the microglial population after ischemic stroke in non-chimeric mice. Infiltration of GFP^+ ^cells was specific to the infarcted cortex, since no increase was observed in the contralateral hemisphere of BM-chimeric mice or in unmanipulated BM-chimeric mice. Other factors contributing to the expansion of the microglial population 12 and 24 hours after pMCAO might be immigration of microglia from regions of the brain not included in the preparation used for flow cytometry, or microglial proliferation. However, microglial proliferation is not prominent at 12 and 24 hours, but is first evident at 48 and 72 hours after induction of focal cerebral ischemia [34], a finding that is similar to observations in other models of acute neural injury [32,36,37].

The use of a GFP BM-chimeric approach in our study of pMCAO served two purposes: 1) direct visualization of infiltrating GFP^+ ^cells in tissue sections and 2) validation of the BM origin of the CD11b^+^CD45^high ^macrophages/granulocytes identified by flow cytometry in our model. In addition to the massive infiltration of GFP^+ ^macrophages/granulocytes 24 hours after pMCAO, we found that a small proportion of microglia in the infarcted cortex could be classified as GFP^+ ^CD11b^+^CD45^dim ^microglial cells. These observations confirm findings by others [38-42], showing a lesion-induced recruitment of microglial progenitors of bone marrow origin into the infarcted cortex. Recently it has been suggested that these microglial progenitors would not enter into the bloodstream or cross the blood-brain barrier under normal physiological conditions [43,44]. However, BM-derived cells have been reported to infiltrate non-irradiated normal brain [45-47], and CD45^high ^leukocytes are routinely detected in unmanipulated, perfused brains by flow cytometry [24,27]. Although focal cerebral ischemia disrupts the blood brain barrier [33,39], and irradiation in itself preconditions the brain for cells to infiltrate the neuropil [43,44], we in line with earlier findings [44] observed only sporadic GFP^+ ^cells in contralateral, non-ischemic cortex and in the brains of unmanipulated BM-chimeric mice. Furthermore, numbers of CD11b^+^CD45^high ^macrophages/granulocytes recruited to ischemic cortex 24 hours after pMCAO were not different between BM-chimeric mice and non-irradiated mice. This suggests that any damage induced by irradiation alone was insufficient to trigger excessive entry of BM-derived cells into the CNS.

Our observation of a significant reduction in microglial population in the cortex of unmanipulated BM-chimeric mice compared to unmanipulated non-chimeric mice indicates that irradiation might impair microglial turnover in normal brain. This is supported by observations by Wirenfeldt et al. [24], who reported that microglial numbers were reduced by approximately 30% in unmanipulated contralateral hippocampi of perforant pathway-lesioned BM-chimeric mice. That study also reported a lesion-induced impairment of the mitotic capacity of microglia in BM-chimeric mice [24]. Taken together, we ascribe the reduced microglial numbers in BM-chimeric mice to irradiation damage.

The quantification of BM-derived GFP^+ ^cells was done by use of an approximated stereological approach, and by use of flow cytometry. The results show that approximately 2/3 of GFP^+ ^cells are lost during tissue processing procedures prior to flow cytometric analysis. Since GFP^+ ^cells located in perivascular aggregates were not included in the approximated stereological analysis, cell loss using flow cytometry might be larger than 2/3. However, the consistency in the data shows that flow cytometry is a reliable and robust tool with which to obtain quantitative data on microglia and infiltrating macrophages/granulocytes in brain pathology, as also has been shown in previous studies [24,27]. Observations made by others using BM-chimeric mice have shown that BM cells have the capacity to differentiate into microglia and perivascular cells [24,39,48], as well as non-myeloid cell types such as endothelial cells, pericytes, astrocytes and neurons [38,42,47,49]. However, in our study of mice with 24-hour survival after pMCAO, we observed no evidence of co-expression of GFP with the astroglial marker GFAP, confirming earlier studies [38,49]. Similarly, we find no clear evidence of co-expression of GFP and vWF/CD31, although such co-expression has been previously reported at later times of observation (3 days – 1 month) after induction of ischemia [38,50].

## Conclusion

We show that IL-1β and TNF-α are produced by largely non-overlapping subsets of microglia and macrophages after pMCAO in mice. This observation is indicative of activation of distinct signalling pathways in different subpopulations of microglia and macrophages after ischemic stroke. Resolution of these pathways may further the development of cytokine-based therapies in stroke.

## List of abbreviations

BM: bone marrow; FCS: fetal calf serum; FSC: forward scatter; GFAP: glial fibrillary acidic protein; GFP: green fluorescent protein; GFP-Tg: C57BL/6-Tg(UBC-GFP)30Scha/J; HBSS: Hank's balanced salt solution; IL-1β: interleukin-1beta; MCA: middle cerebral artery; min: minutes; PBS: phosphate buffered saline; PECAM: platelet endothelial cell adhesion molecule-1; PFA: paraformaldehyde; pMCAO: permanent middle cerebral artery occlusion; RT: room temperature; SB: Soerensens phosphate buffer; s.c.: subcutaneous; SSC: side scatter; TBS: tris buffered saline; TNF: tumor necrosis factor; vWF: von Willebrand factor; WT: B6.SJL-Ptprc^a ^Pepc^b^/BoyJ.

## Competing interests

The authors of this manuscript declare that there are no actual or potential conflicts of interest. The authors affirm that there are no financial, personal or other relationships with other people or organizations that have inappropriately influenced or biased their research.

## Authors' contributions

BHC and KL contributed equally to the experimental and data producing part of this paper. BHC did the data analysis and writing of the manuscript whereas KL assisted in editing the manuscript. AAB was involved in the setup of flow cytometry experiments, interpretation of flow cytometry data and editing of the manuscript, FDH was involved in animal experimentation and TH made the LPS induced macrophage cell cultures. BF contributed to the overall design of experiments and assisted in editing the manuscript.
